# Characterization of Stereolithography Printed Soft Tooling for Micro Injection Molding

**DOI:** 10.3390/mi11090819

**Published:** 2020-08-28

**Authors:** Daniel Dempsey, Sean McDonald, Davide Masato, Carol Barry

**Affiliations:** Department of Plastics Engineering, University of Massachusetts Lowell, Lowell, MA 01854, USA; Daniel.dempsey@newbalance.com (D.D.); Sean_McDonald@student.uml.edu (S.M.); davide_masato@uml.edu (D.M.)

**Keywords:** micro injection molding, additive manufacturing, stereolithography, replication

## Abstract

The use of microfeature-enabled devices, such as microfluidic platforms and anti-fouling surfaces, has grown in both potential and application in recent years. Injection molding is an attractive method of manufacturing these devices due to its excellent process throughput and commodity-priced raw materials. Still, the manufacture of micro-structured tooling remains a slow and expensive endeavor. This work investigated the feasibility of utilizing additive manufacturing, specifically a Digital Light Processing (DLP)-based inverted stereolithography process, to produce thermoset polymer-based tooling for micro injection molding. Inserts were created with an array of 100-μm wide micro-features, having different heights and thus aspect ratios. These inserts were molded with high flow polypropylene to investigate print process resolution capabilities, channel replication abilities, and insert wear and longevity. Samples were characterized using contact profilometry as well as optical and scanning electron microscopies. Overall, the inserts exhibited a maximum lifetime of 78 molding cycles and failed by cracking of the entire insert. Damage was observed for the higher aspect ratio features but not the lower aspect ratio features. The effect of the tool material on mold temperature distribution was modeled to analyze the impact of processing and mold design.

## 1. Introduction

Micro-structured surfaces hold the potential to improve the inherent functionality of products ranging from consumer goods to medical devices to adhesive technology. Whether these systems work based on surface energy modification, light wave manipulation, or microfluidic transport, the inclusion of periodic or asymmetric positive and negative microscale features onto the surfaces of products can yield new properties which would not be intrinsic to an unstructured product. Many researchers have worked at integrating these types of technologies into applications that will have daily use, with a significant focus on surfaces which are inherently super-hydrophobic [1], antireflective [2], and antimicrobial [3]. Researchers also are developing new classes of products, with an example being microfluidic platforms. In general, actual products utilizing surface engineering techniques are somewhat limited, and specifically, the commercialization of microfluidic devices has remained challenging. Issues have been a lack of standards for device testing and development [4], general market dynamics being unfavorable for investment [5], and practices which use processes and techniques for device creation which, at the laboratory scale, are simple and straightforward operations yet ultimately are not feasible to scale for mass manufacturing methods [6].

Table 1 lists techniques and processes which are commonly utilized to produce microfluidic platforms, both on the laboratory scale and for possible commercial mass manufacturing. Devices have been machined directly using micromachining and laser machining techniques, but the minimum feature sizes typically are of around 100 µm [7,8]. While casting of polydimethylsiloxane (PDMS)—i.e., soft lithography—requires low temperatures and pressures, the workflow utilizes labor-intensive techniques, making the process more suitable for laboratory scale development of devices. Hot embossing, roller imprinting, and injection molding provide higher throughputs but require higher temperatures (*T*) and pressures (*P*), as well as greater capital investment. Additionally, these higher throughput workflows often require a series of expensive and time-consuming techniques for the creation of the tooling with micro-structured surfaces [6]. If the development bottlenecks in the higher throughput processes could be solved, such that more iteration could be done on the laboratory scale, this would be an excellent path forward for eventual ease of commercialization.

In injection molding of microfluidic devices, a tooling insert with the microchannel layout is commonly employed in the cavity to form the features on the part surface [11,12,13]. After the predetermined amount of polymer is injected into the mold, pack pressure applied to the melt typically completes the formation of the features. To attain higher replication of microfeatures, the process usually requires elevated melt temperatures [13,14,15] and high injection pressures and velocities [16] to decrease the viscosity of the polymer through shear heating and to delay the solidification of the melt as it comes into contact with the mold wall [15]. Additionally, many researchers believe that mold temperature is the highest temperature setting effect on the process and recommend the mold temperature be higher than the glass transition temperature of the polymer being injected [14,16]. The high temperatures and pressures, however, place considerable stress on the tooling inserts.

When considering the manufacturing processes utilized for the production of microfeature-containing injection molding inserts, a change in design or design complexity is a complicated and expensive endeavor. Over the last 20+ years, many researchers have investigated a variety of materials to employ for tooling inserts in the microinjection molding process, utilizing a wide array of manufacturing techniques [17]. Among the most prevalent in research are silicon produced with x-ray, e-beam, and UV lithography [18], SU-8 epoxy formed via UV lithography [19], hybrid tooling approaches of imprinted polyimide with metalized surfaces [20], electroformed and chemically-etched nickel [21], micro-milled [22], and chemically-etched stainless steel [23], and micro-milled and cast bulk metallic glass [6]. Each combination of tooling material and manufacturing process yields varying capabilities for minimum feature resolutions, lifecycle capabilities, and required lead times for tooling production. Table 2 shows a comparison of these production methods with a view to production scalability.

Current tooling methods fall into one of two categories: (1) fast to produce but limited cycle capability and (2) long lead time for production but high cycle capacity. Further exacerbating the issue is the production method, where each long lead time tool is produced via a process that traditionally makes a single part at a time. Accordingly, scaling a process becomes difficult as parallelization of production requires a high number of tooling inserts. Moreover, for all production methods, a change in design or a desire to evaluate multiple designs becomes problematic as either the window to evaluate is small (low cycle tools) or the lead time to evaluate is large (high cycle tools). Zhang et al. concluded that the more robust materials, such as stainless steel, nickel, and bulk metallic glass, were preferable for molding but encountered challenges from a tool manufacturing standpoint [6]. Resolution limits prevent the use of stainless steel [12,23], void formation in electroformed nickel limits cycles [24], and overall mechanical properties and cost could prevent full adoption of bulk metallic glass [6]. Therefore, the capability to quickly produce tooling inserts with sufficient mechanical properties to withstand the elevated temperatures and pressures of micro injection molding becomes a critical factor to efficiently prototype and scale products.

Additive manufacturing offers the opportunity to merge ease of design changes with an agile manufacturing process. Table 3 outlines the various additive manufacturing technologies broken down by substrate material and process type. When investigating the additive production for injection molding inserts, intuitively, one would gravitate towards metal printing methods. Indeed, there is prior art for researchers utilizing metal printing commonly for the production of inserts with conformal cooling channels [25,26]. At the microscale, however, commercial metal printing processes prove challenging. Generally, metal processes are inhibited by two factors: (1) limited capable resolution due to minimum spot diameters of lasers [27] and (2) high surface roughness due to powder particle size [28].

When Vasco and Pouzada [29] utilized electron beam melting (EBM) to print an injection molding insert containing positive and negative micro walls (200 µm high by 100 µm wide) arrayed in pentagon and star patterns, they noted incomplete feature printing due to the minimum spot size of the laser. This limitation is shared by all of the laser-based metal printing processes as they exhibit limited laser focus diameters of 50–300 µm [27]. After molding with PP, Vasco and Pouzada [29] also reported prevalent stair-stepping on part sidewalls due to printing stratification. Furthermore, Mendible et al. [28] evaluated the direct metal laser sintering (DMLS) process to print bronze casting mold inserts for injection molding and observed a high degree of surface roughness (40–60 µm) when compared to a traditionally machined version. Nagahanumaiah et al. [30] showed that polishing a DMLS bronze tool (for a macroscale gear hub) would allow for thousands of molding cycles in nylon with no tool wear; however, polishing techniques would need to be evaluated for positive tooling microfeatures because the gear hub had a contoured surface with no projections.

While commercial metal printing processes are certainly limited by total resolution for micro-manufacturing applications, polymer processes are split into two groups: (1) print methods which share resolution limits with metal counterparts and (2) processes with acceptable resolution but insufficient material properties. Selective laser sintering (SLS) and multi-jet fusion (MJF) are two examples where resolution limitations are similar to EBM and DMLS; surface roughness can be challenging as typical particle sizes range from 15 to 150 µm [31]. Resolution challenges also hamper fused deposition modeling (FDM), where the print stratification effects and minimum resolution are both a function of the filament diameter deposited by the printer, often limited to a minimum resolution of 100 µm [32]. Furthermore, the filament deposition methods leave voids between perimeter and infill passes, which are not conducive to the necessary compression strength required for injection mold tooling.

For systems which do exhibit acceptable process resolution, material properties often are the challenge. Material jetting (PolyJet) would be an attractive technology, with resolution limits around 80 µm and smooth surface finishes [33]; however, when utilized in studies to create short-run injection tooling, the material heat deflection temperature (HDT) becomes a liability, with all materials exhibiting HDTs under 95 °C [34]. For a PolyJet insert, Mendible et al. [28] reported drastically increased cycle times compared to a traditional machined insert—i.e., 200 and 45 s, respectively—dimensional instability in molded parts within 10 cycles, and tooling life of 130 shots before complete failure. VAT photopolymerization processes show promise in terms of resolution with laser-based SLA systems and DLP-based SLA systems, reporting minimum features of 76 [34] and 19 µm [35], respectively. The differences in X-Y resolution in SLA are due to the choice of print engines: (1) laser-based resolution is defined by the laser spot size, and (2) DLP-based resolution is a function of the pixel size of the projector. For SLA, material development is more advanced than with filament or powder-based methodologies, and some materials have higher heat deflection temperatures (HDT) and flexural modulus properties than PolyJet formulations. When Gheisari et al. [36] employed a DLP-based SLA system to create three tooling inserts for microcantilevers with thicknesses of 30, 120, and 275 µm, they observed failure across three inserts all within five injection molding cycles with polypropylene. They also did note an incorrect print orientation, which may have led to over cure and thus a deterioration in mechanical properties. Vasco and Pouzada [29] also produced star and pentagon featured tooling through a laser-based SLA system, yielding a spot size of 75 µm; initial results were affected by early breakage of microfeatures during the injection cycle, although tool duration was not reported and investigation of wear properties for the SLA tooling was abandoned. Their channel designs had a higher high aspect ratio (AR = 2:1), which could have contributed to stress on the tooling.

The objective of this work was to investigate the feasibility of creating a lower aspect ratio microfeature-enabled tooling for the injection molding process using commercially available additive manufacturing technologies. A DLP-based inverted stereolithography system was utilized to print tooling inserts with an array of 100-μm wide positive microchannels with heights of 24, 47, 71, and 94 µm (creating respective aspect ratios of 0.7, 0.59, 0.39, 0.2) on the surface. Simple microfluidic channels were replicated in high flow polypropylene, and tooling wear was evaluated using a combination of contact profilometry, optical microscopy, and scanning electron microscopy. Tooling life was investigated by molding samples until the tooling fractured during injection molding.

## 2. Materials and Methods

### 2.1. Microstructured Tooling Design

**Figure 1:**
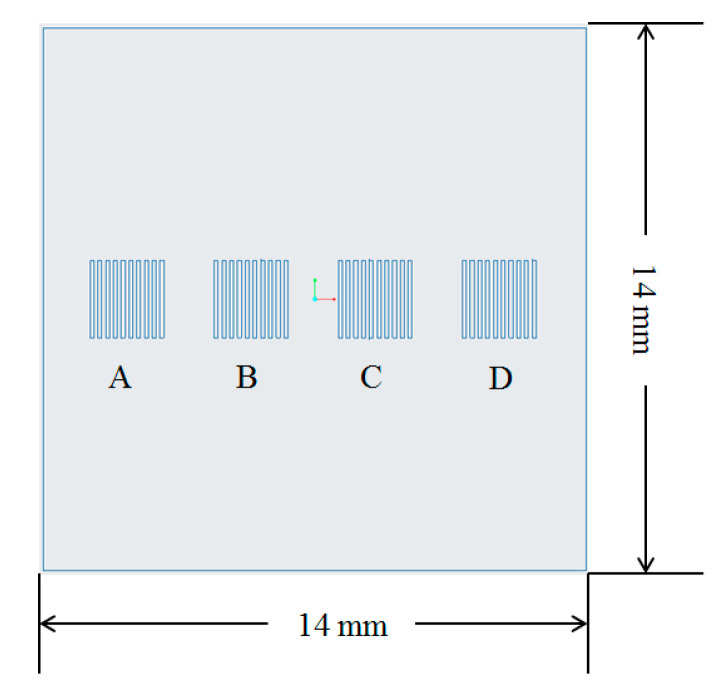
Design of the tooling insert and positioning of the microfeatures.

The micro cavities in the injection molding tooling insert were designed to mimic the features that characterize a microfluidic chip. Figure 1 shows the design of one of the tooling inserts and the location of the microfeatures on its top surface. Different microstructures were designed with different sizes and aspect ratios. Each group of features had a nominal channel width and pitch of 100 µm. However, the structures in the tooling inserts had a height of (A) 25 µm, (B) 50 µm, (C) 75 µm, and (D) 100 µm. Indeed, variation in features height allowed evaluation of the effect of aspect ratio on replication, where researchers identified this as the most significant parameter for replication of microstructured surfaces by injection molding [37]. The tooling inserts had a 14 mm square overall size, with thickness of 10 mm.

### 2.2. 3D Printing of Tooling Inserts

The tooling inserts were produced via inverted stereolithography on a commercially available system (Perfactory 4 Mini, EnvisionTEC GMBH, Gladbeck, Germany). The machine has a maximum build size of 38 × 24 × 220 mm, pixel size of 19 μm, and a *z*-axis resolution of 15–150 μm. In this work, an inverted SLA was selected over traditional SLA because it allows (i) use of resins with improved mechanical properties and (ii) cheaper production with smaller vat requirements [38].

**Figure 2:**
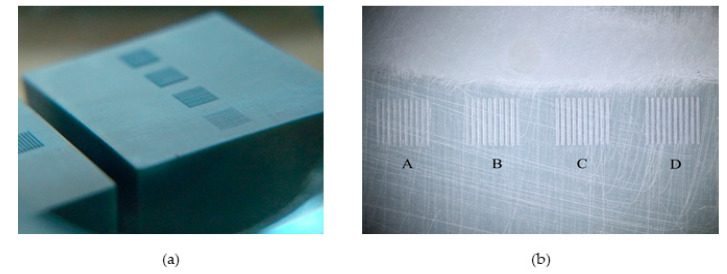
Photos of stereolithography (SLA) 3D printed tooling inserts: (**a**) bulk of the insert and surface microfeatures and (**b**) top view of the printed microfeatures.

The print time for all tooling inserts was 4 h and 12 min, and each insert was UV flood post-cured for 4.5 min. Figure 2 shows one of the printed tooling inserts and details of the microfeatures. A total of 10 inserts were produced for this study; all inserts were printed simultaneously in a single build. This parallelization of tooling production is considered an advantage of the DLP-SLA system.

The resin used to print the inserts was a cross-linkable acrylic-epoxy formulation (HTM 140 V3), whose mechanical properties are reported in Table 4. This formulation was chosen because of its high heat deflection temperature and flexural modulus and the ability to print at a layer thickness of 15 µm. The mechanical properties of HTM 140 V3 suggested its suitability for withstanding the higher pressures and temperatures in the micro injection molding environment.

### 2.3. Injection Molding Setup

**Figure 3:**
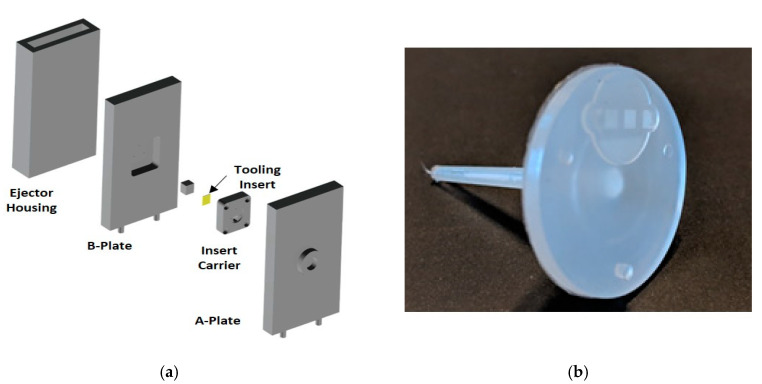
(**a**) Exploded view of the mold base shows the assembly of the 3D printed tooling insert in the steel mold and (**b**) plastic part injection molded using the 3D printed tooling insert and replicated microfeatures.

The tooling inserts printed using SLA were mounted into a mold base using a steel carrier insert. A support pillar and two sheets of 0.4-mm thick polytetrafluoroethylene (PTFE) were used to prevent deflection of the 3D printed tooling insert due to high pressure from the melt polymer. Figure 3a shows an exploded view of the mold base and assembly of the 3D printed tooling insert, which was mounted into the B-plate with the features facing the A-side of the mold. The rest of the mold geometry was machined into the steel B-plate of the mold. The mold was used to manufacture a plastic part with the geometry shown in Figure 3b. The part is a center-gated disk with a thickness of 2 mm and a diameter of 25 mm. The microstructured area was offset to one side of the sprue to provide for parallel flow across the microfeatures. The polymer utilized was a high flow polypropylene (Pinnacle Polymers, Pinnacle PP 1345Z, Garyville, LA, USA), which has a melt flow rate (MFR) of 45 g/10 min.

Injection molding trials were carried out using a 3-ton Nissei micro injection molding machine (Model: AU3E). The machine is characterized by a two-stage injection unit with a 14-mm diameter metering screw and an 8-mm diameter injection plunger. Table 5 reports the main injection molding processing conditions, which were kept constant for all experiments. In particular, temperatures were selected according to indications from resin manufacturer; injection velocity and velocity switchover point were optimized through previous work [12]; packing conditions and cooling conditions were adjusted to eliminate sink mark formation.

The main goal of the injection molding trials was the evaluation of tool life for the SLA 3D printed tooling inserts. Table 6 reports the plan for the injection molding experiments that were carried out using the 10 SLA printed tooling inserts. The evaluation cadence was broken up to yield a view of before and after molding insert conditions and in-process part replication performance across an increasing number of cycles.

### 2.4. Characterization of Replicated Topography

Scanning electron microscopy (SEM, JEOL JSM-7401F, Akishima, Tokyo, Japan) was initially utilized for a qualitative evaluation of microchannels replication. For SEM analyses, samples were sputter-coated with a 10-Å thick layer of gold to permit greater electronegativity. SLA printed tooling inserts were also observed before and post injection molding experiments using confocal microscopy (Zeiss, Stemi 2000 CS, Oberkochen, Germany).

The replication accuracy of the injection molding process carried out with the SLA printed tooling inserts was evaluated by metrological characterization of the replicated structures and comparison with their nominal tool dimension. The height of the replicated microstructures was acquired using a contact profilometer (Bruker XT, Billerica, MA, USA). The height of the replicated structures was measured by monitoring the force transmittance of a stylus as it was dragged across the topography of the sample. The difference between the top surface of the plastic part and the bottom of the structures replicated on the tooling insert was defined as the replicated depth. From channel depth measurements, the feature depth ratio (*DR*) was calculated as

(1) $$DR=\frac{d_{p}}{d_{t}}$$

where *d_p_* is the average depth of the replicated channel in the plastic part, and *d_t_* is the average measured height of the tooling projection.

## 3. Modeling

The injection molding process with the SLA mold was modeled using Moldex3D (CoreTech System Co., Hsinchu County, Taiwan) Studio R17 to understand the effect of the 3D printed mold material on processing. The numerical simulation solves the following system of governing equations to determine the characteristics of the polymer flow [39]. The system includes the conservation of mass (Equation (2)), of the linear momentum (Equation (3)), and of energy (Equation (4)):

(2) $$\frac{d}{dt}\rho +\nabla \cdot \left(\rho v\right)=0$$

(3) $$\rho \frac{d}{dt}v=\rho g-\nabla P+\eta \nabla^{2}v$$

(4) $$\rho c_{p}\left(\frac{\partial }{\partial t}T+v\nabla T\right)=\beta T\left(\frac{\partial }{\partial t}P+\vec{v}\times \vec{\nabla }P\right)+\nabla \cdot \left(k\nabla T\right)+\eta {\dot{\gamma }}^{2}$$

where *ρ* is the density, *η* the melt viscosity, *c_p_* is the specific heat, *β* is the heat expansion coefficient, *k* is the thermal conductivity, *t* is the time, *v* is the velocity vector, *g* is the gravitational acceleration constant, *P* is the hydrostatic pressure, *T* is the temperature, and $\dot{\gamma }$ is the shear rate. Moldex3D solves the governing equations using the high performance finite volume method (HPFVM), which is used to subdivide the computational domain into a finite number of non-overlapping control volumes. For each control volume, the transport variables are calculated at the center of the element; then, the upwind scheme is used to approximate the variables at the faces [40].

The aim of injection molding simulation was that of understanding temperature distribution during the filling of the microstructures. Indeed, when using a plastic 3D printed mold, it is crucial to understand the effect of the lower mold thermal conductivity on the injection molding process. The injection molding simulations were run designing different models with different mold material properties. In particular, the 3D printed tool was compared to a steel and an aluminum tool. Table 7 lists the main properties of the mold materials that were used for the analysis.

**Figure 4:**
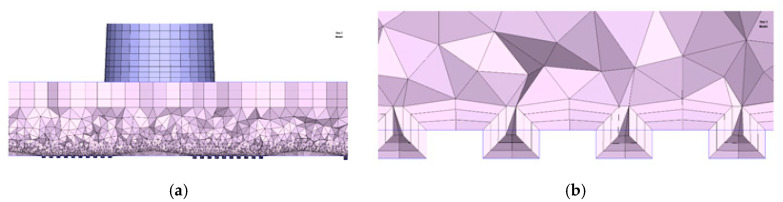
Meshing of (**a**) the part and (**b**) details of the elements in the microstructures.

The CAD model of the part with the microstructures was imported into Moldex3D and meshed using a boundary layer method (BLM) approach (Figure 4a). Seeding was initially carried out to divide the surface of the model with a global edge length of 0.8 mm. Then, the edges of the microstructures were selected to define a smaller element edge size, i.e., 0.025 mm. This allowed having four elements along the thickness of the smaller surface features (Figure 4b). The volume mesh was then created by combining prism elements for the outer layer and tetrahedron in the core of the part. The mold base CAD model was imported and meshed using 3D tetrahedron elements. A mesh size of 2 mm was selected for the mold base, with smaller elements at the interface with the part. The convection between the mold and the environment (i.e., external thermal boundary condition) was defined by specifying an air temperature of 25 °C. The thermal boundary condition at the part/tool interface was automatically calculated by the proprietary software algorithm, which considers the process parameters, material properties, and model geometry.

The polypropylene material database was selected from the software library and used to model the melt polymer flow. Processing conditions were set according to experimental information and machine limitations.

## 4. Experimental Results

### 4.1. Dimensional Characterization of 3D Printed Tooling Inserts

Table 8 presents the dimensions of the micro-structured tooling inserts printed using the inverted SLA process. The dimensions obtained in the tooling were on average 19% wider and 6% shorter than the nominal design dimensions. Thus, the aspect ratio of the microfeatures in the tooling inserts was on average 20% smaller than the design specifications. This deviation in the printed geometry in inverted SLA printing was attributed to two factors: (1) overexposure and compression of initial layers, which causes shortened Z-direction dimensions and (2) the resolution limits of the system chosen for production. When using an inverted SLA process, the first few layers are overexposed to ensure that there is excellent adhesion to the build plate as the part is printed. This overexposure causes the initial layers to be thinner than the standard layer thickness, which has the effect of offsetting all Z height measurements by the compression amount [41].

Regarding the wider-than-designed channels, the increase of 19 µm falls within the resolution of a voxel for the Perfactory Mini system with a 75 mm lens and well within the accuracy of the system. The overgrowth of channels could be eliminated by performing a calibration to the print engine, ensuring perfect alignment with the pixels of the projector, or, at worst, scaling down future geometry to anticipate the XY growth in printing, although pixel growth may still be likely.

### 4.2. Topography Characterization of 3D Printed Tooling Inserts

**Figure 5:**
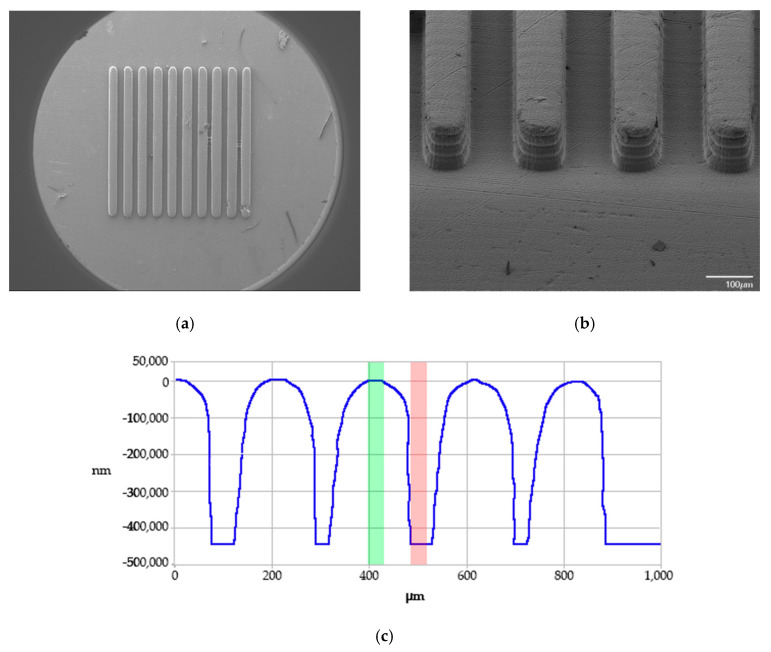
Group B tooling: (**a**) SEM image of the top of features, (**b**) SEM image of isometric view of features, and (**c**) contact profilometry trace of these features.

**Figure 6:**
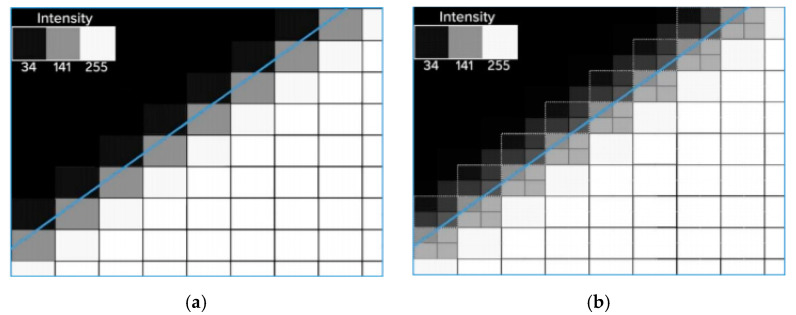
Graphical representation of (**a**) traditional layer-wise stratification and (**b**) DLP anti-aliasing strategy [42].

As illustrated by the scanning electron microscopy (SEM) images and contact profilometry in Figure 5, the overall quality of the printed channels was excellent, with near full fidelity of the channel depths. The qualitative SEM analysis indicated that the 3D printed microstructures were regular and homogenous across the whole printing area, with the absence of significant surface and morphological defects (Figure 5a,b). It is interesting to note that the contact profilometry trace (Figure 5c) indicates that the tops of the channels exhibited a slight rounding effect. This factor was not unexpected as ERM (EnvisonTEC’s Enhanced Resolution Mode), which physically shifts the print engine by one half pixel to essentially deposit two layers in place of one on the part surface, was not used for this work. At the micrometer scale, merely utilizing the printer’s anti-aliasing function was enough to create rounded channel tops. Similar to applications in 2D printing, in the DLP system, surface voxels are exposed on a gradient of 255 shades, with 255 being pure white and 0 being black [42]. The variations in exposure energy lead to a smooth surface finish and, in this work, a rounded microchannel. A graphical comparison of traditional layer-wise stratification, which produces steps in the microfeatures and the anti-aliasing effect that created the rounded features is presented in Figure 6. Some rounding also may have been due to limitations of the contact profilometry measurements.

### 4.3. Injection Molding Process Optimization

**Figure 7:**
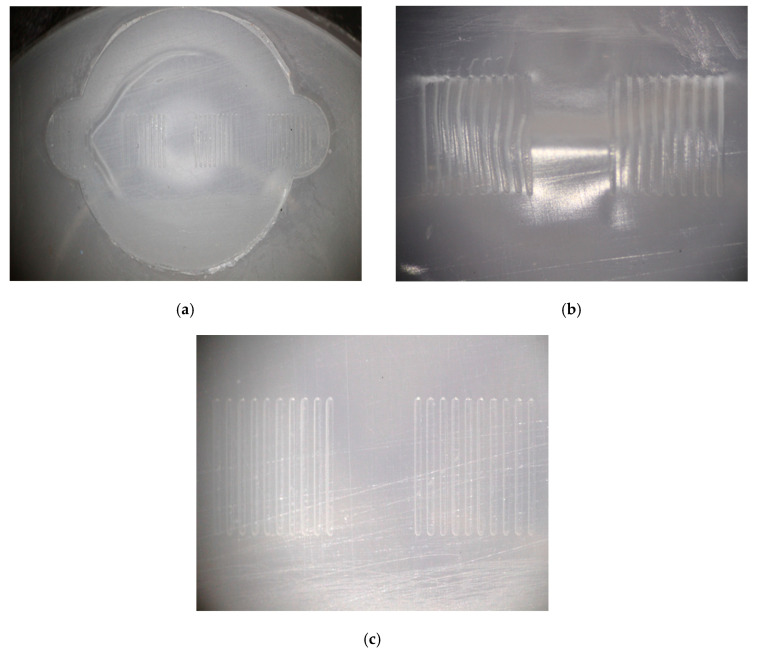
Optical micrographs of (**a**) microfeature molded surface, (**b**) Groups B and C molded with initial processing conditions that produced sink marks, and (**c**) Groups B and C molded with optimized process conditions, which eliminated the sink marks.

The use of 3D printed tooling inserts with microfeatures required process optimization before the tool life for the different inserts could be characterized. Indeed, when molding parts with microstructured surfaces, semi-crystalline materials like polypropylene exhibit higher and more unpredictable levels of shrinkage than amorphous polymers such as polymethylmethacrylate (PMMA) and polycarbonate (PC) [43,44]. The initial trials were run with a barrel temperature of 215 °C, a packing pressure of 10 MPa, and a pack time of 10 s. As shown in Figure 7a,b, these parts exhibited sink marks on the microfeatured surface and the part surface. These sink marks distorted the molded microfeatures (Figure 7b). Elimination of the sink mark required two rounds of process optimization. First, the melt temperature was lowered from 215 °C to 195 °C to reduce shrinkage of the polypropylene melt; this molding was performed with Tool 1. Second, the pack pressure and pack time were increased from 10 MPa and 10 s to 20 MPa and 15 s, respectively, to allow greater compensation for shrinkage. The sink marks were not present with these trials (which were performed with Tool 2). Parts molded with Tools 3, 4, and 5 employed the optimized injection molding conditions and also exhibited no sink marks. Figure 7c illustrates Groups B and C for a Tool 4 part, confirming that process changes had eliminated the defect.

### 4.4. Characterization of 3D Printed Tool Life

**Figure 8:**
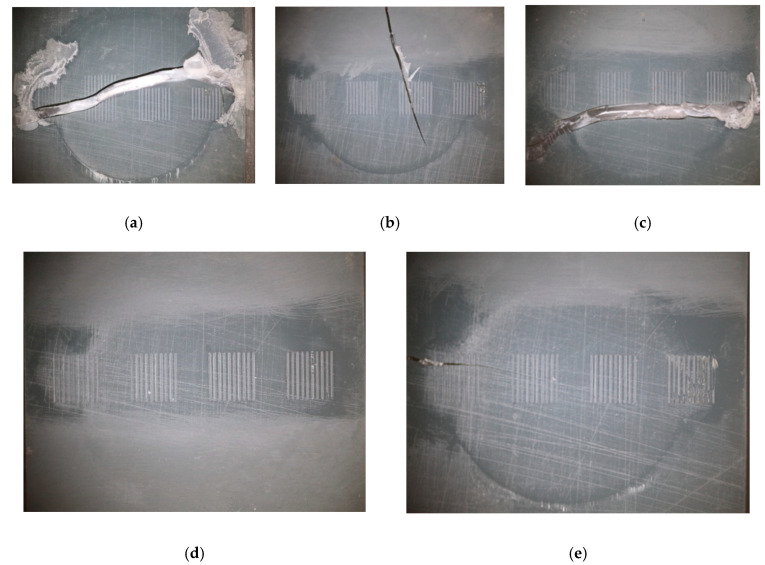
Images of tooling inserts: (**a**) Tool 1 after 36 shots, (**b**) Tool 2 after 16 shots, (**c**) Tool 3 after 29 shots, (**d**) Tool 4 after 60 shots (not cracked), and (**e**) Tool 5 after 78 shots.

The life of the tooling inserts was analyzed using optical imaging of the 3D printed parts at different intervals during the injection molding trials. Different failure behavior was observed, either for the whole insert or for the surface microfeatures. Figure 8 shows images of different failure modes in the 3D printed tooling inserts.

**Figure 9:**
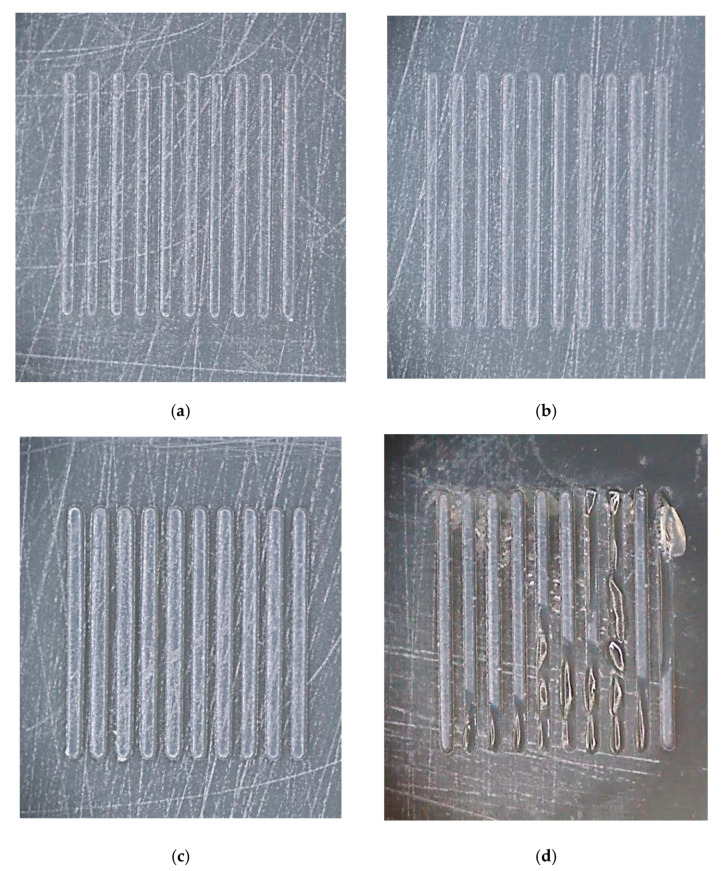
Optical micrographs of intact microfeatures in (**a**) Group A, (**b**) Group B, and (**c**) Group C on Tool 4 after 60 shots, and (**d**) fractured Group D microfeatures on Tool 5 after 78 shots.

During the optimization of the process settings, the inserts proved to be more brittle than anticipated. Tool 1 failed at 36 shots, whereas Tools 2 and 3, which were exposed to higher pack pressures for longer times, failed at 16 and 29 shots, respectively. As shown in Figure 9a–c, the microfeatures in these parts generally did not fail, but rather the entire inserts cracked across the center. With optimized processing conditions, Tool 4 lasted 60 shots without failure and was pulled for tool wear evaluation. Tool 5 failed after 78 molding cycles.

Figure 9d presents a close-up view of Group D features, which had the highest aspect ratio (i.e., 0.79). These positive microfeatures on the surface exhibited consistent brittle failure across all trialed samples. In contrast, the microfeatures in Groups A, B, and C, with aspect ratios 0.20, 0.39, and 0.59, respectively, survived the molding process until bulk tooling failure, with no evidence of brittle failure (Figure 9a–c). The results in Figure 9 are also consistent with the brittle failure of higher aspect ratio positive features experienced in prior work with DLP [29] and SLA [36] molds. Overall, the wear of the features suggests that there may be an aspect ratio limit with this tooling system. Further exploration of SLA formulations, with increased flexural modulus, may be required for higher aspect ratio microfeature durability.

**Figure 10:**
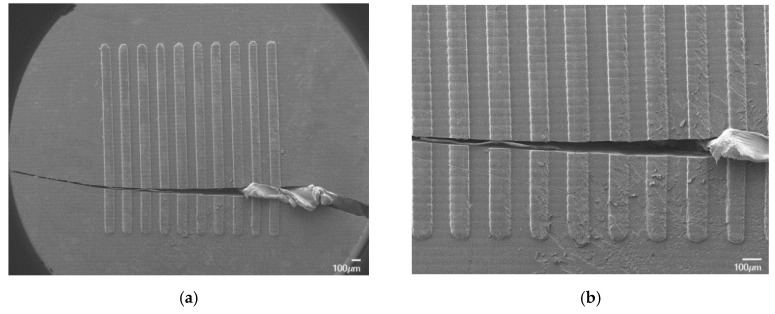
SEM images of Tool 5′s Group D channels exhibiting (**a**) brittle failure and (**b**) thermal-induced surface wear.

The SEM image20 of Group D features from Tool 5 (Figure 10) does indicate some surface deterioration, which can be attributed to thermal wear and failure during ejection. Group D structures failed because of their aspect ratio (i.e., 0.79), which leads to higher and weaker replicated microstructures that are broken during the ejection phase [45]. This is accentuated by the high melt temperature (i.e., 195 °C), which, during the process, is above the HDT for the tooling insert (i.e., 140 °C).

These results, along with the short tool life for Tools 1–3, indicate that the melt temperature and induced pressure on the tooling can affect the long-term durability of both the bulk insert and the microfeatures on the surface. One way to increase the longevity of higher aspect ratio features would be to metalize the surface or use electroplating or other metallization techniques, which could increase the HDT and the heat transfer at the part surface. Additionally, longer tool life may be possible by utilizing a higher strength, higher HDT photopolymer formulation.

### 4.5. Microfeature Replication

Replication of polypropylene microfeature dimensions and shape was acquired through contact profilometry. The average depth ratios (DR) for feature Groups A–D were 99.1 ± 0.4%, 97.8 ± 0.7%, 95.4 ± 1.8%, and 98.6 ± 1.0%, respectively. The total average depth ratio (DR) for all feature groupings was 97.7 ± 1.6%, indicating good replication with this tooling. In this case, aspect ratio had little effect on microfeature replication because all features exhibited high depth ratio values. This finding was not unexpected as the features were relatively wide (119 μm) and had relatively low aspect ratios (<1:1). Moreover, the projections in the tooling surfaces did not hinder the flow of the polymer melt.

**Figure 11:**
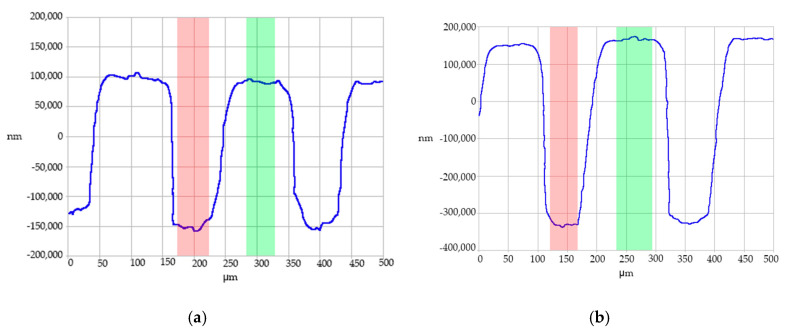

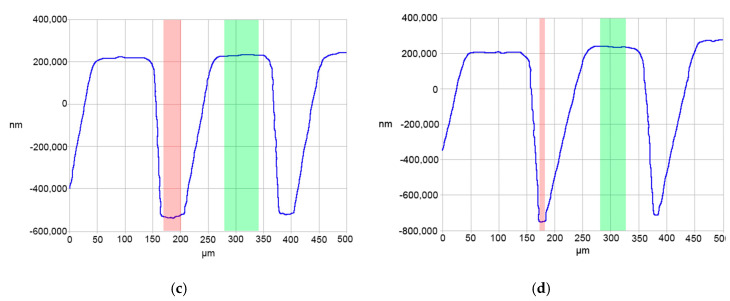
Contact profilometry traces for (**a**) part 45 from Group A, (**b**) part 30 from Group B, (**c**) part 25 from Group C, and (**d**) part 5 from Group D microfeatures molded from Tool 4.

**Figure 12:**
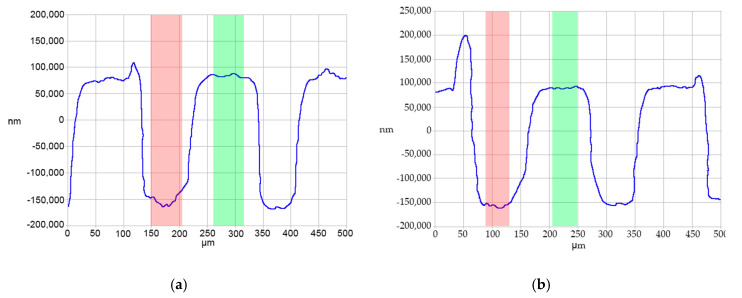
Contact profilometry traces of damaged Group A channels from Tool 4 parts (**a**) 25 and (**b**) 30.

Contact profilometry traces, shown in Figure 11, illustrate the replication fidelity. The profilometry traces were taken from a cross-section of the parts molded from Tool 4. These traces indicate that the lands of the “channels” (which were molded from the base of the tooling) were flat and at a uniform height. The bottoms of the channels were rounded, reflecting the curvature at the top of the projections in the tooling. The shape of the microfeatures also was consistent over multiple molding cycles. Overall, these characteristics indicate a well-replicated system. It should be noted that the contact profilometry exhibited some limitations in the acquisition of the side walls of the microstructures. Indeed, the vertical resolution does not allow accurate reconstructions of the features, as observed in Figure 11 and Figure 12. This limitation, however, does not affect the vertical resolution of the structures that was used as the response variable for the study (cf. Equation (1)).

While the line scans in Figure 12 were indicative of most measured parts, a few traces suggested damage to the Group A features during ejection. The overall severity of the peaks in these parts varied, as did their existence in general. Prior research found similar defects in molded microfeatures and attributed them to demolding issues where the polymer adheres to the feature wall during part ejection [46,47].

## 5. Simulation Results

**Figure 13:**
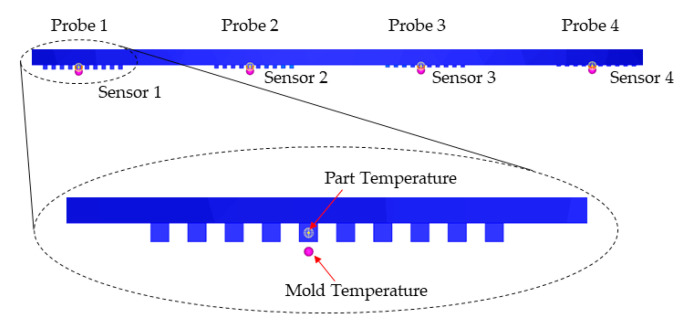
Probe and sensor nodes for monitoring part and mold temperature in the microfeatures.

The numerical model developed in Section 3 was used to study the effect of selecting different mold materials on the injection molding process. For each one of the selected mold materials, a complete Fill + Pack + Cool (Transient) + Warp analysis was run. This approach allowed studying of the transient thermal behavior of the mold. In fact, the thermal analysis is run before the injection starts, in order to determine the steady-state condition, and then during the cycle in order to evaluate the temperature evolution during the process. The temperature of the part and the mold were monitored in the microstructured area for a single cycle, as shown in Figure 13. For each group of microstructures, a probe was inserted to acquire the temperature of the plastic at the entrance of the microstructures. For each group of microstructures in the mold, a sensor was used to acquire the temperature of the tooling at a distance of 0.1 mm from the bottom of each microfeature cavity. This allowed the evaluation of the effect of the aspect ratio on the microscale cavity depth.

The results of the analysis are reported in Table 9. Analyzing the part and mold temperature at different stages of the molding process indicated a stable temperature in the part during filling and permitted evaluation of the effect of microstructures’ positions during cooling. The results show that the different mold material has a negligible effect on the temperature during filling. The differences, however, became significant when considering the temperature distribution during cooling. The tool temperature during cooling was significantly affected by the mold material. In particular, the low thermal conductivity of the 3D printed tool results in higher part temperatures, leading to more difficult heat transfer to the mold base. The slower cooling with the 3D printed tool also was accentuated by the higher mold temperature, which results in increased wear for the microfeatures in the tool. The temperature distribution was also less homogeneous compared to the aluminum and steel tools, in which the probes have reported similar temperature values.

In the SLA 3D printed plastic, the reduced heat transfer promoted the increase of temperature for microfeatures in groups B and C. These feature groups had significantly higher part temperatures during cooling due to the increased difficulty of removing heat from the center of the part. The higher temperature resulted in higher stiction during the ejection phase and thus in easier breakage of the microstructures in the tool. This breakage was more evident for higher aspect ratio features, which have a higher contact area with the mold surface. When considering the mold temperature, the different aspect ratio had no effect on the mold temperature measured at a distance of 0.1 mm from the bottom of the micro cavity.

**Figure 14:**
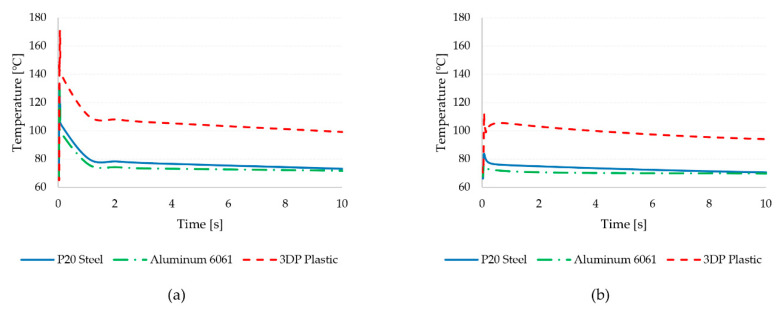
Part (**a**) and mold (**b**) temperature probed close to the microfeatures of Group C for the different mold insert materials (cf. Table 7).

Figure 14 shows the history of temperature in the part and the mold, as measured close to the microfeatures. It can be observed that the metal tools are characterized by a significantly smaller increase in part temperature during filling (Figure 14a). Conversely, the temperature of the polymer increases steeply with the 3D printed tool, and it does not recover after filling upon cooling. The effect of the low thermal conductivity is also visible when considering the mold temperature in Figure 14b. Indeed, the plastic tool leads to a stronger rise in the mold temperature, which is not recovered after filling. These results suggest that the short life of the 3D printed tools can be explained the long time required to stabilize the mold temperature after the cycle. The time required to reach the set mold temperature, however, would require a very long time, thus increasing the cycle time significantly. Moreover, the longer time significantly affects the viscosity and stability of the melt (shot) waiting to then be delivered in the next molding cycle.

The reduced tool life well correlates to the convective thermal load applied by the injected polymer and the reduced thermal conductivity of the insert. A numerical model was created using Moldex3D to analyze the temperature distribution during processing and to compare the 3D printed tool performances with those of conventional metallic mold materials. The simulation results indicated a significant rise in part and mold temperature with the plastic mold. This resulted in reduced heat dissipation to the mold base and eventually in the failure of the soft tooling (cf. Figure 9 and Figure 11). The thermal loads were so significant that failure occurred for the bulk insert, before the micro cavities could be worn off by the polymer flow.

Tooling inserts with projections for simple 100-μm wide microfluidic channels with several aspect ratios were printed on a DLP-SLA system and used to injection mold polypropylene parts. The use of the soft tooling for micro injection molding was analyzed, considering polymer replication and tool life. Injection molding trials and the simulation showed that the DLP-SLA were feasible tooling inserts for injection molding of parts with micro-structured surfaces such as microfluidic channels. Firstly, the DLP-SLA system produced the varying height of tooling geometry to an acceptable deviation from the design, with an overall XY growth of 19% and a depressed Z dimension of 6%. Secondly, the molded parts exhibited high overall replication, with an average depth ratio of 97.7 ± 1.6%. Feature definition also was excellent, with flatlands and rounded channel bottoms in the molded parts as well as relatively smooth part walls. Thirdly, once an optimized injection molding process was established, tooling insert life was 78 shots without bulk failure of the insert. Consistent damage to the tooling projects with the highest aspect ratio (0.79) indicated a minimal aspect ratio for the tooling material. Brittle failure was the dominant failure mode for most inserts.

Modeling of the micro injection molding process with different tool materials allowed for an understanding of the effect of the 3D printed plastic tool. The thermal behavior of the micro-structured tool was observed to be very different compared to conventional metals used for injection molds. The low thermal conductivity of the plastic hinders the heat flow from the part to the mold. This leads to a high thermal load for the microstructures, which fail after repeated cycling. The thermal wear was more accentuated for microstructures with higher aspect ratio, which are more difficult to cool. This indicates the importance of mold design on the wear resistance of the 3D printed tool. Further work will focus on the study of the part/mold thermal boundary condition and its effect on the evolution of the tooling temperature cycle after cycle.

## Figures and Tables

**Table 1:** Comparison of manufacturing processes for micro-featured device manufacturing.

Process	Resolution (µm)	Cycle (min)	Throughput (pcs/mo.)	T (°C)	P (MPa)	Ref.
CNC machining	100	5–30	300–2000	--	--	[7]
Laser machining	100	5–30	300–2000	--	--	[7]
PDMS casting	<1	30–60	150–300	100	0.1	[9]
Hot embossing	<1	10–30	300–1000	100–250	5	[9]
Roller imprinting	<1	0.15–0.5	5000+	100	1	[10]
Injection molding	<1	0.15–0.5	5000+	100–250	10–30+	[7]

**Table 2:** Comparison of technologies for the manufacturing of micro-structured injection molds (adapted from [6]).

Tool Material	Common Manufacturing Method	Minimum Resolution (µm)	Manufacturing Time	Tool Life (Cycles)
SU8	UV Lithography	25	0.5 h	Low (<100)
Silicon	UV Lithography	25	4.5 h	Low (<100)
Hybrid Tooling	Embossing	25	2 days	20–50
Stainless Steel	Micro Milling, Chemical Etching	25	5 days	50,000+
Nickel	Electroforming	5	1–2 weeks	Low (1000)
Bulk Metallic Glass	Micro Milling, Casting	25	5 days	20,000

**Table 3:** Commercial additive manufacturing processes and resolution limits.

Substrate Material	Additive Manufacturing Process	Minimum Resolution (µm)	Refs.
Polymer Processes	Stereolithography	75	[34,35]
	Digital Light Processing	15	[34,35]
	Continuous Digital Light Processing	75	[34,35]
	Fused Deposition Modeling	100	[32]
	Material Jetting	80	[33]
	Multi Jet Fusion	50–150	[27,28]
	Selective Laser Sintering	50–300	
Metal Processes	Direct Metal Laser Sintering	50–300	
	Electron Beam Melting	50–300	
	Laser Engineering Net Shape	50–300	
	Electron Beam Additive Manufacturing	50–300	

**Table 4:** Main properties of the resin selected to print the micro molding tooling inserts [28].

Property	Units	Value
Tensile Strength	MPa	56
Elongation at Break	%	3.5
Flexural Strength	MPa	115
Flexural Modulus	MPa	3350
Heat Deflection Temperature	°C	140

**Table 5:** Process molding conditions adopted for the experimental campaign.

Parameter	Unit	Value
Mold Temperature	°C	65
Barrel Temperature	°C	195
Shot Size	mm	12.5
Injection Velocity	mm/s	80
Pack Pressure	MPa	20
Pack Time	s	15
V/P Switchover Point	MPa	15
Cooling Time	s	10

**Table 6:** Injection molding experimental plan for 3D printed tooling inserts life investigation.

Tool	Cycle Evaluation	Part Measurement	Tool Measurement
		(Cycles)	(Cycles)
1	Process Optimization	-	-
2	20	20	20
3	40	20, 40	40
4	60	20, 40, 60	60
5	80	20, 40, 60, 80	80
6	100	20, 40, 60, 80, 100	100
7	200	50, 100, 150, 200	200
8	300	75, 150, 225,300	300
9	500	100, 200, 300, 400, 500	500
10	1000	200, 400, 600, 800, 1000	1000

**Table 7:** Main properties of the different mold materials used for modeling.

Property	Unit	3DP Plastic	Aluminum 6061	P20 Steel
Density	g/cm3	1.3	2.7	7.8
Heat Capacity	J(KgK)	1030	896	462
Thermal Conductivity	W/(mK)	0.3	154	29
Elastic Modulus	Pa	3.4 × 10^3^	6.9 × 10^10^	2.1 × 10^11^
Poisson Ratio	-	0.35	0.33	0.30
Coefficient of Linear Thermal Expansion	1/K	3.0 × 10^−4^	2.2 × 10^−5^	1.3 × 10^−5^

**Table 8:** Characterization of microfeatures’ dimensions on 3D printed tooling inserts and comparison with nominal design dimensions.

Group	Nominal Dimensions			Measured Dimensions		
	Width (µm)	Height (µm)	Aspect Ratio	Width (µm)	Height (µm)	Aspect Ratio
A	100	25	0.25	119	23.5	0.20
B	100	50	0.50	119	47.0	0.39
C	100	75	0.75	119	70.5	0.59
D	100	100	1.00	119	94.0	0.79

**Table 9:** Maximum temperature values for the part and the mold monitored for the different mold materials.

	P20 Steel			Aluminum 6061			3DP Plastic		
	Part		Mold	Part		Mold	Part		Mold
	Fill/Pack	Cooling		Fill/Pack	Cooling		Fill/Pack	Cooling	
A	193.0	82.0	80.1	192.9	75.8	75.4	193.6	110.3	99.4
B	194.7	133.2	83.3	194.5	130.0	76.7	194.9	170.2	101.8
C	195.1	165.0	84.2	195.1	163.5	77.5	195.2	185.3	100.3
D	194.8	86.5	84.0	194.8	78.0	77.1	194.5	101.9	99.8
Average	194.4	116.7	82.9	194.4	111.8	76.7	194.5	141.9	100.3
Dev. Std.	0.9	39.7	1.9	1.0	42.6	0.9	0.7	42.0	1.1
Max.	195.1	165.0	84.2	195.1	163.5	77.5	195.2	185.3	101.8
